# Does adding a psychosocial cessation intervention to an existing life-skills and tobacco-prevention program influence the use of tobacco and supari among secondary school students?: Findings from a quasi-experimental trial in Mumbai, India

**DOI:** 10.18332/tpc/113355

**Published:** 2019-11-26

**Authors:** Nilesh Chatterjee, Himanshu Gupte, Gauri Mandal, Tshering Bhutia

**Affiliations:** 1 Salaam Bombay Foundation, Mumbai, India; 2 Narotam Sekhsaria Foundation, Mumbai, India

**Keywords:** tobacco, adolescents, supari (betel nut), psychosocial cessation intervention, school-based

## Abstract

**INTRODUCTION:**

This study aimed to test whether school-going adolescents who self-report tobacco and/or supari use are more likely to quit if a school-based psychosocial cessation intervention is added to an existing life-skills and tobacco-prevention program.

**METHODS:**

A quasi-experimental trial with pre-test and post-test 20 weeks after the intervention was conducted with students from low-income families in 12 schools in Mumbai; six schools were randomly assigned to the intervention and the remaining to the comparison condition. Participants were students from grades 7, 8 and 9 who self-reported tobacco and/or supari use. Intervention schools received six sessions of LifeFirst, a psychosocial group-based tobacco cessation intervention program, in addition to SuperArmy, a school-wide life-skills and tobacco-prevention program. Trained counselors facilitated the cessation intervention, which spanned five months. All students in comparison schools received only SuperArmy. The outcome measures were self-reported use of tobacco-only, supari-only, and tobacco plus supari in the past 30 days.

**RESULTS:**

The number of all users decreased by 19.1% in the intervention and 18.7% in the comparison schools at post-test. Although this reduction was significant (p<0.001) within each group, the difference between intervention and comparison schools was not significant. Further segregation by type of product used showed that for tobacco-only users there was a non-significant increase of 1.7% in intervention schools, and a significant 26.2% increase (p<0.001) in the comparison group. Tobacco plus supari use declined in both groups; however, supari-only use fell by 14.8% in the intervention and 32.7% in the comparison schools (p<0.01).

**CONCLUSIONS:**

The combination of a cessation intervention along with the life-skills and tobacco-prevention program appear to have halted tobacco-only use in the intervention group. Future research needs to determine whether students are substituting supari for tobacco and to understand the psychological mechanisms underlying the cessation intervention and the interaction between cessation and prevention-only interventions.

## INTRODUCTION

India has nearly 266 million tobacco users^1^,^2^. Tobacco use is associated with a million deaths in the country^3^ and two in five (40%) of all-cancer related deaths and 90% of all oral cancer deaths^4^,^5^. Tobacco-related morbidity and mortality in the country generate an economic burden of about US$ 22.4 billion^6^.

Adolescence is a critical and susceptible phase for initiating tobacco-use; four in ten tobacco users in India start before the age of 18 years^7,8^. According to the Global Adult Tobacco Survey, as of 2016–2017, 13% of youth aged 15–24 years use tobacco^2^. The 2009 Global Youth Tobacco Survey (GYTS) found that the prevalence of smoking among boys aged 13–15 years, was almost three times higher than that among girls; and that 45% of these male adolescent users had initiated bidi (a type of local cigarette) smoking before the age of 10^7,9^. The use of supari, also known as areca nut or betel nut, is also high among children and adolescents, who are often oblivious to its deleterious health effects^10,11^. Supari is one of the most widely consumed addictive substances in India after nicotine, ethanol, and caffeine. In the context of the state of Maharashtra, in which the city of Mumbai is located, supari use is perceived as culturally acceptable by many social groups and often consumed during festivals^11^. Supari has been reported to cause or exacerbate conditions such as myocardial infarction, hepatotoxicity, obesity, type II diabetes, and asthma, and is a risk factor for cancers of the mouth and esophagus^12^. In India, many smokeless tobacco products contain areca nut as an ingredient; and various sweetened preparations containing areca nut are sold as ‘flavored supari’ in colorful, attractive sachets for children; supari consumption often begins at a young age^13,14^.

The risks of tobacco-related diseases are highest for those who start early and continue its use for a long period^15^. With 40% of India’s population^16^ under the age of 19 years, efforts targeted at curbing tobacco and supari use among adolescents are critical to protect this vulnerable group from addiction and reduce the burden of tobacco-related morbidity and mortality. Tobacco prevention policies and programs have been found to be effective in preventing initiation among adolescents and youth^17,18^. In India, the Cigarettes and Other Tobacco Products Act (COTPA), enacted in 2003, provides directives for the regulation of trade and commerce, advertising, production, supply and distribution of cigarettes and other tobacco products. Two of its provisions focus exclusively on the adolescent age group, viz the ban on the sale of tobacco products to and by persons aged below 18 years, and the prohibition on the sale of tobacco products within 100 yards of all educational institutions^19^. In 2009, the Ministry of Health and Family Welfare released comprehensive guidelines for achieving tobacco-free schools and educational institutions^7^. The Central Board of Secondary Education took this initiative a step further by setting 11 criteria for a tobacco-free school, which include prevention-related activities in schools^20^.

Recognizing the importance of de-addiction efforts along with prevention and education, tobacco cessation clinics that offer counseling, medication, and nicotine replacement therapy were established by the Ministry of Health and Family Welfare^21,22^. In 2016, the m-Tobacco Cessation Program, a mobile-phone-based intervention, and a national toll-free quitline were launched by the Indian government. As of 2017, this program has 20 million registered users with a 7% quit rate among smoked and smokeless tobacco users^23,24^. However, no such cessation programs have been designed exclusively for adolescents in the country despite data from the GYTS of 2009 revealing that two-thirds (66%) of adolescents who smoked tobacco wanted to stop smoking and 67% had tried to quit^7,8^. Schools can improve student tobacco quit rates by providing effective cessation assistance to their students and staff who use tobacco^25^; however, in India, apart from a few sporadic interventions by leading tobacco control organizations, there are no reported country-wide or state-wide tobacco cessation programs targeting adolescents. Furthermore, while the Indian government has designed policies to regulate the sale and distribution of tobacco, especially among children, through COTPA 2003, there are no such restraints on the sale of supari to minors.

The present study was conducted with secondary school students who self-reported tobacco and/or supari use. It assessed whether a school-based group psychosocial intervention program for the cessation of tobacco and supari use offered to self-reported users along with an existing classroom-based life-skills and tobacco-prevention program for all students was more likely to influence existing users to quit compared to users in schools that received only the classroom-based life-skills and prevention program.

## METHODS

### Study setting and participants

This study was conducted in 12 schools directly managed and financially aided by the Brihan-Mumbai Municipal Corporation (BMC), the local government of the city of Mumbai, Maharashtra. BMC schools serve students from low-income families and have a uniform structure of management, type of teachers, and curriculum. These schools, with the support of Salaam Bombay Foundation, a non-government organization (NGO), also provide secondary school students a classroom-based life-skills and tobacco-prevention program entitled SuperArmy.

### Study design and participants

A quasi-experimental trial was conducted from August 2015 to June 2016 wherein 12 BMC-affiliated schools of similar profiles were chosen for the study. Students in all 12 schools were already recipients of a classroom-based life-skills and tobacco-prevention program entitled SuperArmy. Of these, six schools were randomly selected for the intervention condition; all self-reported tobacco and supari users received an additional psychosocial tobacco/supari cessation intervention entitled LifeFirst, which was offered in specially designed sessions for smaller groups of users and delivered in a separate designated space in the intervention schools. It is important to note that students in the intervention schools also received the SuperArmy life-skills and prevention program in their respective classrooms along with their non-user classmates. Students, both tobacco/supari users and non-users, in the remaining six schools, assigned to the comparison condition, only received the SuperArmy program in their regular classrooms.

Change in tobacco and/or supari use was assessed by comparing the status of use at the baseline conducted before the start of the intervention, and a post-test conducted around 20 weeks after the end of the intervention. The intervention sessions were spread over 5 months; however, the period between the baseline and the post-test was around 11 months.

### Data collection

A structured questionnaire captured data on: i) sociodemographic details of participants including gender, age, grade; ii) recent use of tobacco and/or supari in the past seven days through the items ‘Have you used any form of tobacco in the past seven days?’ and ‘Have you used supari in the past seven days?’; iii) recent use of tobacco and/or supari in the past 30 days through the items ‘Have you used any form of tobacco in the past 30 days?’ (including questions on different forms of tobacco) and ‘Have you used supari in the past 30 days?’; iv) ever use of tobacco and/or supari; v) knowledge, attitudes and beliefs related to tobacco and supari through items such as ‘Is there any association between health problems and tobacco use?’, ‘Smoking is a cool behavior’, ‘Is it possible to purchase any supari product within 100 yards of school?’, and ‘How easy will it be for you to turn down a smoking request made by your best friend?’.

Participating students from each school completed the questionnaire in their respective classrooms during school hours. No school teacher or any instructor connected to the school-based tobacco interventions was present in the classroom during the administration of the questionnaire. Trained research facilitators, recruited specifically for this study and not known to the students, read out the questionnaire in the local language of Hindi or Marathi as per the requirement of each school. After the facilitator read out each question, respondents marked their responses to the questionnaire. The researchers decided to use this method over a completely self-administered survey given the comprehension level of the students. Facilitators were trained in rapport establishment with children, standardized techniques of reading and explaining questions to the students, maintaining the confidentiality of respondents and scrutinizing filled in instruments for completeness.

The study was approved by the Institutional Ethical Review Board of Narotam Sekhsaria Foundation and Salaam Bombay Foundation. Written consent for the study was sought from school principals. Parents of the students were also informed about the scope of the study and gave written consent before the recruitment of their children in the study. Additionally, student assent was sought before responding to the questionnaire.

### Interventions

#### LifeFirst cessation program

The LifeFirst tobacco and supari cessation program was developed by the Salaam Bombay Foundation and Narotam Sekhsaria Foundation to support tobacco and supari users to quit. This school-based program was adapted for the adolescent age group and the curriculum was validated by psychology experts. The intervention consisted of six group sessions, each lasting 50 minutes. Participants were students who self-reported tobacco and/or supari use in the baseline survey and expressed interest in quitting. These students were called to a separate room in the school and group sessions were held during the school day. Trained counselors facilitated the sessions that were held about two weeks apart. A total of six sessions were completed in the six intervention schools over a period of five months.

Session one consisted of an introduction and team-building activities. Session two raised awareness about the ill-effects of tobacco and supari and explored patterns of tobacco/supari use among students. Session three explored obstacles to quitting such as triggers, craving, and withdrawal, and ways to overcome them. Session four focused on skill-development for tobacco/supari refusal including assertiveness and examined the causes of relapse among students. In session five, students were recognized and appreciated for their efforts to quit tobacco/supari use, and refusal and assertiveness skills were further practiced. Session six conducted a review of all the lessons in the LifeFirst program, discussing refusal and relapse prevention in detail.

#### SuperArmy life-skills and tobacco-prevention program

The SuperArmy program of the Salaam Bombay Foundation is designed to build awareness about the harmful effects of tobacco, develop life-skills needed to refuse tobacco and empower students to become advocates for change in their communities. This three-year program starting in the 7th grade begins by introducing students to the concepts of addiction and peer pressure and builds life-skills like confidence, communication, and refusal skills. In the second phase, students learn advocacy skills and engage with stakeholders like the police, government officials, school administrators, and tobacco vendors. Students work towards making their schools and communities tobacco-free and in the process continue to develop life-skills such as teamwork and leadership.

### Outcome variables

The primary outcome variable was self-reported tobacco and/or supari use, with recent use measured through dichotomous Yes/No responses to the following items: ‘In the last 30 days have you smoked cigarettes, smoked bidis, smoked hukkah, used chaini or khaini, mawa, loose tobacco with chunaa, or tobacco pan?’. Anyone who answered Yes to any of these questions was considered a recent tobacco user. Recent supari use was determined through responses to the following question: ‘In the last 30 days have you used supari?’. Anyone who answered Yes was considered a recent supari user. Those who answered Yes to any form of tobacco use, as well as supari use, were considered recent tobacco and supari users. Abstinence from tobacco/supari was not verified by any other means.

### Data analysis

Trained staff entered the data from the completed questionnaires into an MS-Excel sheet. The data were cleaned and then analyzed in Stata Version 15.1^26^. Only those students who had completed surveys at the baseline and post-test were included in the analysis. Descriptive statistics were generated for all the variables. Changes in proportions of recent users between time points were assessed using a proportions analysis for each arm separately. To assess the difference between arms, a logistic regression was conducted to control for baseline use (tobacco only, supari only, or both tobacco and supari), gender, age and number of sessions attended. For all analyses, a p-value of <0.05 was considered statistically significant.

## RESULTS

Surveys were conducted among students in grades 7, 8 and 9 from 12 participating schools; with 570 students in the intervention schools and 638 students in the comparison schools completing both the baseline and post-test at 20 weeks. Analysis of the baseline survey for all students, who completed both baseline and post-test surveys, showed that students in the intervention schools were more likely to be younger, with a significantly higher proportion (33.7%) in the age group 10–12 years, compared to 26.5% aged 10–12 years in the comparison group (p<0.05). However, the comparison group had a significantly greater number of female students (356; 55.8%) than the intervention group (279; 48.9%) (p<0.05).

A significantly greater proportion of students in the comparison schools (55.8%) reported that it was possible to purchase tobacco within 100 yards of the school premises than students in the intervention group (45%) (p<0.01). Similarly, a significantly higher proportion of comparison school students (56.8%) answered in the affirmative for the item ‘possible to purchase supari within 100 yards of school premises’, compared to the intervention schools (44.7%) (p<0.01). However, the number of students who were aware of the health consequences of tobacco, although not statistically significant, was higher in the intervention group (67.8%) than in the comparison group (63.4%). A very small number of students in both intervention and comparison schools believed that using tobacco and supari were cool behaviors, or that tobacco and supari use relieved stress. About one in five students from both, the intervention group (19.3%) and the comparison group (20.2%) reported difficulty in turning down an offer to smoke by a best friend. Close to a fifth of intervention group students (18.1%) and comparison group students (18.9%) reported difficulty in refusing a best friend’s offer to use supari.

In the baseline survey, 115 students from the intervention schools and 107 students from the comparison schools self-reported any form of tobacco and/or supari use in the past. Analysis of the self-reported users from both groups (Table 1) revealed that the intervention group had a greater proportion of males (80%) and students in grade 9 in contrast to the comparison schools. However, there were no statistically significant differences in age, grade or gender of the students in intervention and comparison groups.

**Table 1:** Comparison of baseline characteristics, including tobacco-related knowledge and attitudes, for all students (irrespective of tobacco and supari use) and students self-reporting use in intervention and comparison schools (only those who completed both baseline and post-test surveys are included in this table)

*Variables*	*Baseline for all students, irrespective of use, who completed both surveys*			*Baseline for self-reported users who completed both surveys*		
	*Intervention group (n=570) n (%)*	*Comparison group (n=638) n (%)*	*p^a^*	*Intervention group (n=115) n (%)*	*Comparison group (n=107) n (%)*	*p^a^*
**Age^b^** (years)			**0.020**			0.467
10–12	192 (33.7)	169 (26.5)		30 (26.1)	27 (25.2)	
13–15	361 (63.3)	445 (69.7)		75 (65.2)	74 (69.2)	
16–18	17 (3.0)	21 (3.3)		10 (8.7)	5 (4.7)	
**Gender^b^**			**0.036**			0.120
Male	290 (50.9)	282 (44.2)		92 (80.0)	76 (71.0)	
Female	279 (48.9)	356 (55.8)		23 (20.0)	31 (29.0)	
**Grade**			0.717			0.081
7th	117 (20.5)	120 (18.8)		23 (20.0)	16 (15.0)	
8th	204 (35.8)	228 (35.7)		32 (27.8)	45 (42.1)	
9th	249 (43.7)	290 (45.5)		60 (52.2)	46 (43.0)	
**Knowledge, attitudes and beliefs (only those who gave affirmative responses to statements)^c^**						
Smoking is a cool behavior	5 (0.9)	5 (0.8)	0.753	3 (2.6)	4 (3.8)	0.876
Use of smokeless tobacco is a cool behavior	4 (0.7)	6 (0.9)	0.367	1 (0.9)	2 (1.9)	0.801
Use of supari is a cool behaviour	17 (3.0)	15 (2.4)	0.583	12 (10.5)	11 (10.4)	0.997
Smoking makes people free from stress and makes them comfortable	10 (2.5)	11 (2.3)	0.876	5 (6.7)	5 (5.9)	0.838
Smokeless tobacco makes people free from stress and makes them comfortable	6 (1.5)	7 (1.5)	0.965	3 (3.7)	3 (3.8)	0.988
Supari makes people free from stress and makes them comfortable	9 (1.6)	6 (0.9)	0.587	7 (6.3)	2 (1.9)	0.275
**Knowledge, attitudes and beliefs (only those who gave affirmative responses to statements)^c^**						
People who use tobacco have more friends	59 (10.4)	62 (9.8)	0.649	18 (15.9)	16 (15.1)	0.904
People who use supari have more friends	53 (9.3)	59 (9.3)	0.934	19 (16.7)	18 (17.0)	0.816
It is possible to purchase tobacco within 100 yards of school premises	256 (45.0)	354 (55.8)	**<0.001**	75 (65.2)	70 (67.3)	0.744
It is possible to purchase supari within 100 yards of school premises	254 (44.7)	360 (56.8)	**<0.001**	76 (66.1)	75 (70.8)	0.456
It is not at all easy to turn down a request to smoke, if made by a best friend	109 (19.3)	128 (20.2)	0.500	26 (23.0)	30 (28.3)	0.607
It is not at all easy to turn down a request to use smokeless tobacco, if made by a best friend	97 (17.1)	119 (18.9)	0.542	20 (17.5)	29 (27.6)	0.134
It is not at all easy to turn down a request to use supari, if made by a best friend	103 (18.1)	119 (18.9)	0.643	28 (24.3)	32 (30.5)	0.485
There is an association between health problems and tobacco use	385 (67.8)	400 (63.4)	0.110	77 (67.0)	58 (55.8)	0.089
**Recent use of tobacco and/or supari^d^**						
Recent use of tobacco only (in any form, smoking or smokeless)	7 (6.1)	8 (7.5)	0.680			
Recent use of supari only	71 (61.7)	66 (61.7)	0.993			
Recent use of both tobacco (in any form) and supari	37 (32.2)	33 (30.8)	0.831			

a Chi-squared test for independence of positive, negative and neutral responses, excluding missing values.

b Percentages may not sum to 100% due to missing data for some variables.

c Percentages for non-missing responses only.

d Percentages for those with recent use only in past 30 days.

Tobacco and/or supari users from both the intervention and the comparison group, did not differ significantly with respect to tobacco and supari-related attitudes and beliefs. Very small proportions of users believed that tobacco and supari use constituted cool behavior, or that they relieved stress. A higher proportion of intervention group users (67%) were aware of the adverse health effects of tobacco use compared to comparison group users (55.8%); however, this difference was not statistically significant. Furthermore, there were no significant differences in the numbers of users from intervention and comparison groups who reported that it was possible to buy tobacco or supari within 100 yards of the school premises. Similar proportions of users from the intervention and the comparison groups reported facing difficulty in refusing a best friend’s offer to smoke or use supari.

Before the start of the intervention (at baseline), there were 115 self-reported users of any form of tobacco and/or supari in the intervention schools and 107 in the comparison schools. At follow up in the post-test, there were 93 self-reported users in intervention and 87 in comparison schools; that is the number of users decreased by 19.1% in the intervention group and 18.7% in the comparison schools. Although the reduction was significant within each group, the difference between intervention and comparison schools was not significant. For all types of users combined, the recent use of tobacco and/or supari declined in both groups, though not significantly different from each other.

Further segregation of those who did not quit showed a slightly different picture for the different types of users. Table 2 illustrates this in more detail – showing the percentages of participants at two time points that had recently used tobacco-only, supari-only, or used both tobacco and supari. At baseline, the number of recent tobacco-only users was 7 and 8, respectively, in the intervention and comparison groups; at post-test they were 9 and 36, respectively, a non-significant increase of 1.7% in intervention schools, and a significant 26.2% increase in the comparison group. The number of students reporting the use of both tobacco and supari at baseline was 37 and 33, respectively, in the intervention and comparison groups; at the post-test it was 30 and 20, respectively, a decrease of 6.1% and 12.1%. At the baseline, the intervention group had 71 supari-only users, which decreased to 54 at post-test, a statistically significant reduction of 14.8% (p<0.05) within this group. In the comparison schools, the number of supari-only users was 66 at baseline, which showed a greater reduction of 32.7% to around 31 self-reported users at post-test.

**Table 2:** Recent tobacco and supari use by time point (data at baseline and post-test 2)

*Condition*	*Outcome (recent use, 30 day usage)*	*Baseline*	*Post-test*	*Baseline vs Post-test*	*p-value for difference between intervention and comparison at post-test^c^*
		*Number of recent users (% of condition)*		*Change in % of recent users (p-value)^a^*	
**Intervention group** (n=115)	All users of tobacco and/or supari	115	93 (80.9)	**-19.1 (<0.001)**	0.905
	Tobacco-use only	7 (6.1)	9 (7.8)	+1.7 (0.604)	**<0.001**
	Supari-use only	71 (61.7)	54 (47.0)	**-14.8 (0.024)**	**0.007**
	Use both tobacco and supari	37 (32.2)	30 (26.1)	-6.1 (0.310)	0.202
**Control group** (n=107)	All users of tobacco and/or supari	107	87 (81.3)	**-18.7 (<0.001)**	
	Tobacco-use only	8 (7.5)	36 (33.6)	**+26.2 (<0.001)**	
	Supari-use only	66 (61.7)	31 (29.0)	**-32.7 (<0.001)**	
	Use both tobacco and supari	33 (30.8)	20 (18.7)	**-12.1 (0.040)**	

a Test for difference in proportions (two-tailed).

b Differences-in-differences repeated measures mixed model for differences between conditions.

c Logistic regression controlling for baseline use – tobacco, supari or both.

The changes over time in the comparison and intervention arms are compared in Table 2 (last column). Although there was no significant difference between the conditions for the overall outcome variable of all users, there was a statistically significant difference wherein comparison schools reported increased use of tobacco-only at post-test (p<0.001) compared to intervention schools. However, reduction in supari-only use was greater in comparison schools compared to the intervention schools (p=0.007). Concerning attendance in sessions of the psychosocial cessation intervention, an *ad hoc* sensitivity analysis did not find a significant difference in tobacco and/or supari use reduction for those who attended at least one session in the intervention group, controlling for the number of sessions attended.

## DISCUSSION

The present study, a quasi-experimental trial, tested whether offering a school-based cessation intervention in addition to a classroom-based tobacco-prevention program influenced more tobacco and/or supari users to quit compared to receiving a prevention program only. This study found a decline in supari-only use and tobacco plus supari use in both the intervention and comparison groups. However, while tobacco-only use remained static in the intervention group between the two measurements, the comparison group showed a statistically significant rise in the number of tobacco-only users at post-test. The combination of a cessation intervention along with the life-skills and prevention program seemed to halt tobacco-only use in the intervention group. In the comparison group, which received the tobacco-prevention program of SuperArmy, supari use declined but with little effect on tobacco-only use. The data seem to show that while the tobacco-prevention program does address supari use in both groups, adding the cessation intervention, LifeFirst, appears to have some effect on addressing the tobacco-only use rates in the intervention group. The question is whether the students from the comparison schools moved from supari to tobacco use. Data to prove the exact effect of the SuperArmy program on students substituting products is lacking in this study. The mechanisms by which the SuperArmy program acts on prevention and cessation as well as how the two programs – SuperArmy and LifeFirst – act on tobacco versus supari use need to be examined in more detail. The present study did not examine how the messages received from two different sources, SuperArmy and LifeFirst, were processed by intervention students. Future research will have to examine these processes, and identify and measure key behavioral variables involved in the process of change.

Tobacco use among teenagers remains a problem, however, most tobacco control programs for adolescents are based around the prevention of initiation of smoking or tobacco use^27^. In the Indian context, tobacco prevention and life-skills programs have been implemented for adolescent cohorts, and some have even demonstrated effectiveness in trials^28,29^. However, programs that address initiation might not specifically address the needs of existing adolescent tobacco users. Various tobacco cessation interventions for adolescents have been tested globally, including psychosocial and behavioral approaches (such as school and community-based programs, pharmacotherapy, and tobacco control policies), but these interventions had mixed results^30^. It appears that the most significant therapeutic effect in teenagers was observed for self-monitoring and coping skills, motivational strategies (reducing ambivalence to change), group counselling, and addressing social influences that affect smoking behavior^27,30-33^. The National Tobacco Control Program in India has made provisions for cessation services, a national quitline, and mobile-phone-based cessation support program^24^, yet, tobacco cessation interventions designed specifically for adolescents are few in the country.

The present study is among the few in India to examine the effects of providing a school-based psychosocial cessation intervention for both tobacco and supari. This study used a quasi-experimental design that tested the intervention in a real-world scenario of local government schools that cater to students from low-income backgrounds. The study was pragmatic in that it tested the feasibility and effectiveness of the intervention in the actual day-to-day setting of these schools; it was implemented by actual LifeFirst staff rather than a specialized research and intervention team. The lessons learned from the study, with appropriate contextual and sociocultural adaptation, could be useful to those working on tobacco and supari cessation among adolescents in India.

### Limitations

This study has some limitations. The comparison schools received a life-skills and tobacco-prevention program called SuperArmy, which might also have some effect on cessation. Data collection relied primarily on self-reported tobacco and supari use; this might be a source of error, especially arising from under-reporting of use subsequent to the interventions. The surveys were conducted in classrooms, and although the research facilitator was not known to students, and no school teachers or tobacco-program instructors were present, classroom-based surveys on topics such as tobacco use could give rise to socially desirable responses. The SuperArmy program has a longstanding presence in the participating schools and various school-based activities are conducted by the program staff, and students associate tobacco-related activity with that program. Therefore, although no SuperArmy staff were present during the survey, it is possible that students in comparison schools responded in a manner they considered would be favorable to SuperArmy personnel because of this association. Future studies may be better served if they use a biochemical validation of tobacco use such as the use of salivary cotinine to minimize error from inaccurate reporting. Unmeasured factors may also exist between the intervention and comparison schools that affect the results. Living in a social environment consisting of fewer smokers has been identified as one important predictor of quitting smoking^33^. Family and community-related factors will have to be measured in cessation studies conducted with school students. In the future, the LifeFirst cessation intervention should seek greater understanding of family dynamics of enrolled students and the role of family members.

## CONCLUSIONS

The findings of this study underscore the need to look deeper into the factors that influence tobacco and supari cessation in adolescents as well as the mechanisms by which school-based interventions act on cessation. Supari is emerging as an insidious and persistent hazard to adolescent health in India. This study highlights the need for a deeper examination of the interactions and relationship between supari use and tobacco use in adolescents.
